# Modeling Cell-Free Protein Synthesis Systems—Approaches and Applications

**DOI:** 10.3389/fbioe.2020.584178

**Published:** 2020-10-28

**Authors:** Jan Müller, Martin Siemann-Herzberg, Ralf Takors

**Affiliations:** Institute of Biochemical Engineering, University of Stuttgart, Stuttgart, Germany

**Keywords:** *in vitro* protein synthesis, cell-free synthetic biology, mathematical model, *in silico*, ribosomes, transcription and translation, modeling

## Abstract

*In vitro* systems are ideal setups to investigate the basic principles of biochemical reactions and subsequently the bricks of life. Cell-free protein synthesis (CFPS) systems mimic the transcription and translation processes of whole cells in a controlled environment and allow the detailed study of single components and reaction networks. *In silico* studies of CFPS systems help us to understand interactions and to identify limitations and bottlenecks in those systems. Black-box models laid the foundation for understanding the production and degradation dynamics of macromolecule components such as mRNA, ribosomes, and proteins. Subsequently, more sophisticated models revealed shortages in steps such as translation initiation and tRNA supply and helped to partially overcome these limitations. Currently, the scope of CFPS modeling has broadened to various applications, ranging from the screening of kinetic parameters to the stochastic analysis of liposome-encapsulated CFPS systems and the assessment of energy supply properties in combination with flux balance analysis (FBA).

## Introduction

Cell-free protein synthesis (CFPS) technology has a long history in life sciences, which started with fundamental research on deducing the genetic code (Nirenberg and Matthaei, 1961). Over several decades, the system was developed stepwise into a polypeptide production machinery (Spirin et al., 1988). Since the early adaptations of the system for commercial use (Shimizu et al., 2005), an increasing number of applications have emerged in the market (e.g., PURExpress, PURE*frex*, PURE*frex*2.0, myTXTLkit). These are based either on synthetic transcription–translation systems with a well-defined composition or on crude cell extracts that contain a more complex component assembly. While the product titer and production volume of such systems have increased from a few microliters to hundreds of liters, several limitations of the system remain. To date, and even within the best commercial systems, the protein titer with CFPS systems is orders of magnitude lower than that of *in vivo* whole-cell production due to resource expense and reduced longevity (Carlson et al., 2012; Gregorio et al., 2019; Silverman et al., 2019). Here, purpose-driven modeling can be a crucial tool to push the boundaries forward and identify bottlenecks.

CFPS is an “open” experimental system that allows defined reaction setups, which is ideal for simulation approaches. It has emerged not only as a research tool for the processes of transcription and translation but as a biomanufacturing platform for rapidly prototyping production systems *in silico* and *in vitro* (Laohakunakorn et al., 2020; Vilkhovoy et al., 2020). For a variety of transcriptional and translational components, kinetic parameters are known, allowing the study of their behavior. Yet, one of the most fundamental principles of modeling always restricts the approach; the accuracy of model predictions cannot exceed the granularity of the model itself. In other words, distinctions between experimental observations and simulations are likely to occur if model predictions, extrapolated data sets, or fundamental model structures do not reflect the real problem. Consequently, such discrepancies may motivate a more thorough study of the experimental problem. Hence, proper model design aims to reflect reality with sufficient granularity (e.g., should the maturation of a reporter be considered?), thereby building on a solid mixture of experimentally validated data supported by assumptions. In this regard, we provide a brief overview of the existing models for CFPS and related systems and how they are applied to specific cases. It must be stated that many models have been developed with different objectives regarding the system environment, model approach (deterministic, stochastic), and granularity. Therefore, the models are not categorized as “good” or “bad” but clustered and assessed with respect to their particular purpose. In contrast to the mini-review of Koch et al. (2018), which focuses on deterministic models for CFPS, we expand the scope to adjacent fields and highlight qualitative and quantitative model characteristics.

**FIGURE 1:**
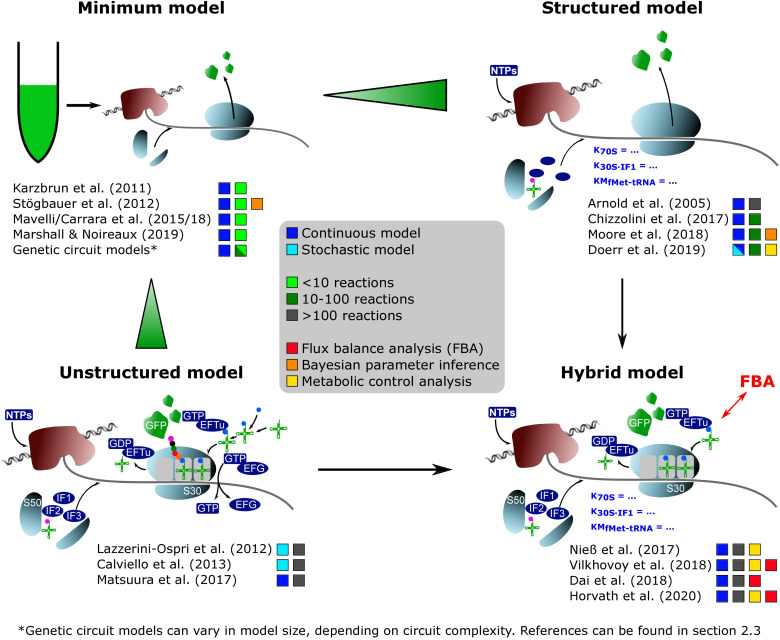
On-trend categorization of CFPS models with respect to the defined model levels “minimum,” “structured,” “unstructured,” and “hybrid.” Color-coded squares indicate model classes, size, and particular features. The transition from “minimum” to “structured” considers the implementation of detailed kinetics. In contrast, the shifting from “minimum” to “unstructured” extends the reaction network and kinetic complexity. “Hybrid model” represents a tradeoff between “structured” and “unstructured” approaches. NTPs, nucleoside triphosphates; S50/S30, ribosomal subunits; EFTu, elongation factor Tu; EFG, elongation factor G; IF1/IF2/IF3, initiation factors; GFP, green fluorescent protein.

When developing a model, it is necessary to know the components that should be considered. A CFPS system typically consists of, at least, the core components of transcription and translation: a mRNA polymerase, ribosomes, translational factors, amino acyl-tRNA synthetases, amino acids, tRNAs, an energy regeneration system, and nucleotides (Shimizu et al., 2005). Additionally, the DNA substrate, the produced mRNA, and the product (in most cases presented here, GFP derivatives) must be considered. If a crude cell extract is used, the system becomes much more complex, as the concentration of many of the components is unknown. The modeling studies on CFPS presented in detail in section “**Development and Application of CFPS Models**” share the common goal of identifying key model parameters by parameter regression on experimental data. However, the complexity of the models differs. By trend, the models may be divided into four groups of different granularities (Figure 1):

**“Minimum model”:** Minimal models, presented in section “Identifying Bottlenecks in CFPS Systems,” take into account up to ten parameters or equations, mainly focusing on macromolecular components such as mRNA and DNA. They are the backbone for more detailed descriptions. Additionally, most of the genetic circuit models presented in section “**Extending the Scope of CFPS Modeling**” can be described as minimum models.

**“Structured model”:** Medium-scale models that introduce structured descriptions of certain aspects of the transcription-translation network. In structured models, kinetic models such as Michaelis–Menten or Hill are implemented in combination with larger ODE systems of up to 100 equations.

**“Unstructured model”:** Large-scale models are fine-grained. They are meant to describe the CFPS in a holistic way and comprise networks of several hundred reactions. These models typically use simple individual reactions without further structural elements in order to save computational costs (e.g., Matsuura et al., 2017).

**“Hybrid model”:** A special case are hybrid models, connecting structured models to other networks such as metabolic networks, or unstructured parts of lumped elements. Intrinsically, the approach increases the model complexity and computational costs. However, it offers an in-depth analysis of CFPS.

## Development and Application of CFPS Models

Continuous models are typically used to simulate CFPS systems. They make use of ordinary differential equations (ODEs) and algebraic equations to dynamically describe model states. In a structured model, equations incorporate affinity constants and other parameters. To reduce complexity, models can be formulated following an unstructured black-box approach, considering only apparent kinetics (Bailey and Ollis, 1986). The differences between the model types are dynamic. Often, models are partly structured to focus on selected segments of the reaction network with particular interest. We call these approaches “hybrid models.” The quality of CFPS mechanistic models relies heavily on the proper model structure and the correct identification of model parameters. Given the complex nature of CFPS, the precondition of independent datasets for parameter identification is challenging and may require repeated careful consideration for each regression analysis (Golightly and Wilkinson, 2005; Moore et al., 2018). Stochastic effects play only a minor role in most of the classical CFPS modeling approaches. In liposome or droplet-based CFPS, due to small reaction volumes, low numbers of molecules may cause rendering reactions between different molecules in stochastic events. Under such conditions, a description with a discrete and stochastic model is preferable (Gillespie, 1977; Frazier et al., 2009).

### Identifying Bottlenecks in CFPS Systems

The most straightforward way of describing *in vitro* expression of GFP is to consider the macromolecular components, DNA, mRNA, and proteins in a black-box model (Karzbrun et al., 2011; Stögbauer et al., 2012; Chizzolini et al., 2017; Marshall and Noireaux, 2019). This allows fitting kinetic equations to the experimental results of GFP production, mRNA production, and mRNA degradation. RNA polymerase and ribosomes are considered as catalytic components. For simplification, we call such approaches “minimum models.” Karzbrun et al. (2011) proposed a coarse-grained dynamic model consisting of four enzymatic reactions. Kinetic studies were performed for crude cytoplasmic extract from *Escherichia coli* to identify biosynthesis and degradation parameters. A similar granularity was chosen by Stögbauer et al. (2012) to simulate and analyze the results gathered with the PURExpress system. Here, the model neglects protein degradation but covers a broader experimental range, identifying the plateau phase when the translational system expires. A comparable ODE-based model was applied to a variety of regulatory elements (promotor strength) to identify limitations in the resources of the transcription–translation system (Marshall and Noireaux, 2019). Here, the commercially available “myTXTLkit” was used. Despite the application of different CFPS systems, all models revealed a saturation effect in GFP production under increased DNA template concentrations. Chizzolini et al. (2017) extended the minimal model to describe the expression of different fluorescence proteins under various regulatory elements. Here, limitations of current CFPS models were addressed, namely the specificity for only narrow experimental data sets, limited prediction capacity, and neglecting biophysical factors (e.g., RNA secondary structure).

With a model system of similar complexity, it was shown that limitations of CFPS may occur, which could not be mirrored by minimum models (Doerr et al., 2019). Using a comprehensive experimental data set of commercial *E. coli* CFPS, depletion of tRNAs and translation initiation were identified as limiting factors. By extending the minimum model to a structured description with additional terms for inactive mRNA states, it was possible to improve the prediction quality for the experimental data.

More fine-grained models were necessary to identify the challenging substrate limitations *in silico*. Such a model was introduced in our laboratory by Arnold et al. (2005) and recently renovated (Nieß et al., 2017). In this hybrid model, a simplified transcriptional model was connected to a detailed description of the translation process. The unique approach uses a ribosome flow model to simulate the movement of the ribosome along a one-dimensional discrete template (MacDonald and Gibbs, 1969; Heinrich and Rapoport, 1980). This approach enables a careful study of the influence of different components on the translation rate. The elongation factor Tu and tRNA concentration were identified as the most sensitive parameters hampering the translation rate. As a key difference between *in vitro* and *in vivo* conditions, a control shift from the ternary complex to translation initiation was identified.

An equally complex system was developed to describe the synthesis of a short Met-Gly-Gly peptide in an *E. coli-*based *in vitro* system by incorporating 968 reactions and 241 components (Matsuura et al., 2017). The approach evaluated the stability of pseudo-steady states, revealing the temporal stability of metabolite clusters, their collapse, and re-merge, until a final steady state is reached. Interestingly, increasing tRNA supply also led to a slight increase in translation rates (observed as increased poly-peptide production), but the effect was much less dominant, as shown by Nieß et al. (2017).

### Analysis and Prediction of Liposome-Encapsulated Protein Synthesis

A special case of *in vitro* protein synthesis is the encapsulation of CFPS components in liposomes. In this model, only a few stochastically distributed components may be balanced, creating different reaction conditions in the vesicle and outside the vesicle. The initial studies showed that GFP production kinetics strongly depend on liposome size and lipid composition (Sunami et al., 2010). In a first attempt to simulate protein synthesis inside liposomes, a medium-sized CFPS model considering 30 species and 106 reactions was connected to a stochastic model for encapsulation (Lazzerini-Ospri et al., 2012). Later, the model was extended to 280 species and 270 reactions, comprising a coupled transcription–translation model (Calviello et al., 2013). The approach allowed screening of GFP production with different start conditions either by looking at different liposome diameters or by considering different quantities of CFPS components inside the liposome. In agreement with continuum CFPS simulations, optimal DNA levels were identified for maximizing GFP formation. Oversaturation of the system with DNA decreased GFP yields. This finding reflected the enormous energy needs for the transcription process. Follow-up studies showed that some of the results could be achieved with a much less complicated model. Here, around 10 reactions were incorporated by lumping reactions for tRNA charging, transcription, translation, and energy regeneration. The simplified model described the behavior of the PURE system under 27 different compositions, rendering resource availability from standard conditions to limitation, remarkably well (Mavelli et al., 2015; Carrara et al., 2018).

### Extending the Scope of CFPS Modeling

CFPS systems have emerged as ideal test beds for genetic circuits, allowing easier and faster prototyping than traditional in-cell engineering. Consequently, mathematical models to describe those systems have been developed (Niederholtmeyer et al., 2015; Takahashi et al., 2015). They cover a wide range of regulatory circuits: two-gene cascades (Siegal-Gaskins et al., 2014), sigma factor guided regulation (Adhikari et al., 2020), complex genetic ring oscillators (Niederholtmeyer et al., 2015), and experimentally verified RNA circuit controllers (Agrawal et al., 2018, 2019; Hu et al., 2018). The developed “minimum models” typically consist of three to ten ODEs and mass balances, considering mRNA, regulatory RNAs, or protein products as model species, and mass action, Hill, or Michaelis–Menten kinetics for regulatory descriptions. Even with coarse-grained models, the highly dynamic systems could be mirrored and predicted successfully. The model-guided circuit design significantly reduced development times.

The use of *in silico* models is not limited to well-defined model systems such as *E. coli* crude extract or commercial products. Moore et al. (2018) broadened their application to study the CFPS capacities of *Bacillus megaterium*, linking robotic liquid handling with a coarse-grained ODE model (26 parameters, 14 species, and 18 reactions) for the TX-TL system. Key kinetic parameters of the xylose-repressor system were approximated from DNA titration experiments. Simulations were performed using parameters identified by Bayesian parameter inference. Extending the model to describe the concurrent expression of two targets, plasmids carrying GFP and mCherry derivatives revealed competition for translational resources. In general, the reported translation elongation rates (between 0.10 and 0.02 aa s^–1^) were slower than those reported for CFPS systems. The inefficient use of available energy accounted for the low performance. Another model approach for investigating resource competition in CFPS was formulated by Gyorgy and Murray (2016) with a minimal model for genetic circuits. Here, the authors could successfully quantify the burden of two targets expressed simultaneously on the resources of a CFPS system.

A constraint-based model to approximate energy and substrate supply from *E. coli* CFPS extract was presented by Varner and colleagues (Dai et al., 2018; Vilkhovoy et al., 2018; Horvath et al., 2020). They connected a simplified description of protein production (Allen and Palsson, 2003) and allosteric enzyme regulation (Wayman et al., 2015) with the metabolic network. Flux balance analysis (FBA) was applied to estimate the flux patterns of central carbon metabolism, amino acid biosynthesis, and energy metabolism using the objective function of maximizing the production rate of chloramphenicol acetyltransferase (CAT). Analysis of different amino acid supply scenarios *in silico* revealed inefficient energy yields of the experimental *in vivo* setup, most likely due to unfavorable side reactions. Similar scenarios may have also occurred in the experimental setup for *B. megaterium* described above.

## Discussion

A historical trend can be observed regarding the objectives of CFPS models. Early models (Arnold et al., 2005; Karzbrun et al., 2011; Stögbauer et al., 2012) are focused on the basic CFPS system using GFP as an experimental readout, beyond classical targets such as ß-galactosidase, chloramphenicol transferase, luciferase, or other likewise “easy to quantify” targets. In the past decade, an increasing number of diverging scientific branches have developed broadening the scope of model building and simulations. Yet, the GFP-based system is described in most detail and is the focus of current investigations. Currently, derivatives of the initial GFP are commonly used, such as its “enhanced” and “super folder” variants (Pédelacq et al., 2006; Shin and Noireaux, 2010). The mRNA product is typically quantified with RNA aptamer reporters such as the malachite green RNA aptamer. The broadening of scientific approaches and the extension of cell-free genetic circuits will increase the need for easy and reliable reporter systems based on short nucleotide or peptide sequences (Wick et al., 2019).

When analyzing the different model granularities (Figure 1), major differences are observed. For coarse-grained models, compromises are made by assuming certain states of the model system by neglecting components or by lumping different metabolites (e.g., all amino acids) to one species. Fine-grained models consider these species in detail. System complexity has been increased from small systems with around 10 equations to large models with hundreds of reaction components (Table 1). Increasing complexity can offer the possibility to resolve bottlenecks by getting insights into reactions or reaction networks. Experimental access to all process elements is hardly possible, and only subsets of information are normally available, even for best investigated bacterial strains such as *E. coli*. As a result, complex models usually rely on multiple data resources covering different experiments (Matsuura et al., 2017; Nieß et al., 2017), whereas small models may be well identified by single experiments. Interestingly, it was shown that results gathered with complex systems can also be mimicked with reduced systems (e.g., Mavelli et al., 2015). Consequently, deciding a proper CFPS model structure should be driven by the questions to be answered, and should critically reflect the database for model identification.

**TABLE 1:** Overview of the different granularities of CFPS models.

**References**	**Features**	**No. of parameters**	**No. of species**	**No. of equations**	**k_TX_ [nt s^–1^]**	**k_TL_ [aa s^–1^]**
Karzbrun et al. (2011)	Minimal model of the CFPS system	10	7	4	0.50	4.00
Stögbauer et al. (2012)	Refined minimal model of the CFPS system	8	5	4	2.20	0.03
Mavelli et al. (2015) and Carrara et al. (2018)	Simplified CFPS model for screening of different CFPS compositions	16	9	6	1.67	0.09
Marshall and Noireaux (2019)	Simulation of different transcription (promoter) and translation initiation (ribosome binding site) configurations	14	5	5	10.00	2.50
Chizzolini et al. (2017)	Study on different fluorescence protein targets, regulatory elements, and critical evaluation of model prediction	12	10	10	–	–
Moore et al. (2018)	Model description and kinetic parameter estimation for CFPS of non-model bacteria	26	14	18	8.13–11.47	0.09–0.11
Doerr et al. (2019)	Identification of translation initiation as bottleneck of CFPS, analysis of different commercial kits	13	10	10	–	–
Lazzerini-Ospri et al. (2012) and Calviello et al. (2013)	*In lipo* protein synthesis, stochastic distribution of components in liposomes	24–280	106–270	–	19.00	4.00
Matsuura et al. (2017)	Quasi-stationary state analysis of complex model networks	483	241	968	–	–
Nieß et al. (2017) (based on Arnold et al., 2005)	First detailed description of coupled transcription and translation model (Arnold). Comparison of *in vitro* and *in vivo* conditions, metabolic control analysis	>70	174 + no. of codons	>500	–	1.12
Dai et al. (2018); Vilkhovoy et al. (2018), and Horvath et al. (2020)	Coupling of CFPS to flux balance network of the central carbon metabolism, implementation of allosteric regulation	–	146	264	–	–

*Each reference is listed with its unique features alongside the number of parameters, species, and equations incorporated in the model. If available, the transcriptional and translational rate constants k_*TX*_ and k_*TL*_ as readouts of the model are given. Models that share the same background are lumped together (note that genetic circuit models are not considered here).*

The quality of CFPS models is checked by challenging model predictions with experimental observations. Typically, rates for transcription (k_TX_), translation, and elongation (k_TL_) are experimental readouts. However, the range of these parameters is broad (Table 1). k_TX_ has been reported from 0.5 (Karzbrun et al., 2011) to 19 nt s^–1^ (Calviello et al., 2013). k_TL_ ranged from 0.03 (Stögbauer et al., 2012) to 4.00 aa s^–1^ (Karzbrun et al., 2011; Calviello et al., 2013). The apparent differences may reflect the intrinsic problem of using relatively few experimental readouts to identify models of different complexities (Chizzolini et al., 2017). It has been shown that even simultaneously planned and performed CFPS experiments can lead to significant outcomes between different laboratory sites (Cole et al., 2019). As the modeling studies presented here are based on a wide range of commercial and homemade CFPS systems and extracts, this might explain the deviance of calculated parameters.

## Conclusion

Currently, CFPS models can identify bottlenecks in the transcriptional and translational processes as well as infer kinetic parameters from model data. The consensus of most model predictions is the identification of the translational rather than the transcriptional process as one of the key targets for further developments in CFPS systems. Potential starting points are translation initiation, tRNA supply, and recycling. In most approaches, the modeled mechanisms of the translational process seem to be oversimplified. Inspiring approaches for *in vivo* translation have been published by Vieira et al. (2016) and Dykeman (2020) that could be adapted to *in vitro* descriptions. For many modeling purposes, hybrid models can be the ideal tradeoff between complexity and acceptable computational costs. As CFPS systems and genetic circuits get more complex and consider multiple targets (RNAs/proteins), models that consider the joint burden on resources will come into focus (Gyorgy and Murray, 2016; Borkowski et al., 2018). A feedback loop between the model investigation and experimental setup has to be established. The works on genetic circuit models have proved that fast and easy prototyping is possible with CFPS. To unravel the key mechanisms for designing models, data from metabolomics and proteomics have to be integrated. Recent research addresses this need and offers a variety of datasets that could be harnessed by the CFPS modeling community (Garcia et al., 2018; Garenne et al., 2019; Miguez et al., 2019). This significantly increases the possibility to describe CFPS with an improved mechanistic resolution, up to a complete dynamic description of the CFPS system components. The development will open the door for a thorough application of tools of statistical systems analysis and metabolic control analysis to translate simulation results into system engineering advice.

## Author Contributions

JM reviewed the literature, designed the concept, wrote the manuscript, and prepared the figures. MS-H and RT co-edited and supervised the manuscript. All authors approved the manuscript for publication.

## Conflict of Interest

The authors declare that the research was conducted in the absence of any commercial or financial relationships that could be construed as a potential conflict of interest.
